# Complete genome sequence of strain KD-1^T^, the type strain of *Sulfurisphaera javensis*, isolated from an Indonesian hot spring

**DOI:** 10.1128/mra.00664-24

**Published:** 2024-10-29

**Authors:** Hiroyuki D. Sakai, Naswandi Nur, Antonius Suwanto, Norio Kurosawa

**Affiliations:** 1 Department of Science and Engineering for Sustainable Innovation, Faculty of Science and Engineering, Soka University, Hachioji, Tokyo, Japan; 2 Research Center for Applied Microbiology, National Research and Innovation Agency, Jl Raya Jakarta-Bogor KM 46, Cibinong, Bogor, West Java, Indonesia; 3 Department of Biology, Faculty of Mathematics and Natural Science, IPB University, Bogor, Indonesia; California State University San Marcos, San Marcos, California, USA

**Keywords:** Archaea, thermophiles, hot springs, Indonesia, *Sulfurisphaera*, chemolithotrophy

## Abstract

*Sulfurisphaera javensis* KD-1^T^, originally isolated from an Indonesian acidic hot spring, is a hyperthermophilic, acidophilic, and facultatively anaerobic archaeon. Here, we report the 2.6 Mb complete genome sequence of KD-1^T^. Genes related to sulfur metabolism were found in the genome, indicating that KD-1^T^ is involved in the sulfur cycle in its habitat.

## ANNOUNCEMENT

*Sulfurisphaera javensis* is a hyperthermophilic, acidophilic, and facultatively anaerobic archaeon originally isolated from an Indonesian acidic hot spring (1). The genus *Sulfurisphaera* belongs to the order *Sulfolobales*, one of the dominant groups inhabiting acidic hot environments. Increasing genomic information on this taxon contributes to our understanding of the genomic adaptation and evolution of microorganisms inhabiting acidic/hot environments (2). Here, we report the complete genome sequence of KD-1^T^.

Hot water was collected at Kawah Domas hot springs and used as an inoculum for enrichment culture using MBSY medium (1) at 75°C. The strain KD-1^T^ was isolated from the enrichment culture as described previously (1). The strain was cultured in 200 mL of MBSY medium at 80°C for 5 days. After centrifugation (15,000 × *g*, 15 min), DNA was extracted from the pellet using Genomic-tips 100/G (QIAGEN). After the DNA library was prepared with the NEB Next Ultra II FS DNA Library Prep Kit for Illumina (New England BioLabs), short-read sequencing was performed on the NovaSeq 6000 platform (150 bp × 2). As a result, a total of 8,304,418 short reads composed of 1,245,662,700 bp were obtained. Default parameters were used for all software unless otherwise specified. The short-reads were quality-filtered using fastp v.0.20.1 (3), resulting in a total of 8,272,862 quality-filtered short-reads composed of 1,240,847,940 bp. Long-read sequencing was carried out using MinION with an R9 flow cell, SQK-LSK109, and EXP-NBD104 (Oxford Nanopore Technologies: ONT), following the protocol described by ONT (NBE_9065_v109_revZ_14Aug2019). Longer DNA fragments (>3 kb) were enriched using the Long Fragment Buffer included in the SQK-LSK109 kit, following the manufacturer’s protocol. As a result, a total of 43,990 long-reads composed of 300,265,271 bp (N50: 12,873 bp) were obtained. Long-reads were quality filtered by Filtlong v.0.2.0. (https://github.com/rrwick/Filtlong) using short-reads as an external reference with the following options: --min_length 1000 --keep_percent 90 --trim --split 100 --mean_q_weight 10. As a result, a total of 36,943 quality filtered long-reads (270,240,902 bp, N50: 10,954 bp) were obtained. Using the quality-filtered short/long-reads, genome assembly was conducted by Unicycler v.0.4.8 (4), followed by annotation with DFAST v.1.4.0 (5). As a result, a circular genome of 2,569,661 bp with a GC content of 31.7%, determined by the Unicycler, was obtained. The sequence coverage calculated by CoverM (https://zenodo.org/records/10531254) was 482×. The genome contained 2,782 CDSs, a single copy of rRNA operon (16S-23S), 46 tRNAs, and 5 CRISPR repeats. The genome completeness and the contamination ratio were assessed at 99.72% and 0.99%, respectively, by CheckM (6). The 16S rRNA gene sequence obtained from the genome showed a 100% sequence identity to that of *S. javensis* KD-1^T^ (accession number: LC332080), as calculated by blastn against the NCBI-nr database. The *rgy*, a key gene for adaptation to high temperatures (7), was found in the genome, consistent with the hyperthermophilic lifestyle of *S. javensis* KD-1^T^ (1). Several genes related to sulfur metabolism, such as *doxAD*, *sor*, *sqr*, *sreABC*, and *soeB*, were found in the genome, indicating *S. javensis* KD-1^T^ is involved in the sulfur cycle in its habitat.

## Data Availability

The genome sequence of strain *S. javensis* KD-1^T^ and raw reads have been deposited in DDBJ/ENA/GenBank under the accession numbers AP031322, DRR537969, and DRR537970.
